# Efectos secundarios de la exposición a metales en la cirugía del *pectus excavatum*

**DOI:** 10.23938/ASSN.1129

**Published:** 2025-09-17

**Authors:** Julio César Moreno-Alfonso, Esther Comajuncosas Pérez, Ada Yessenia Molina Caballero, Alberto Pérez Martínez

**Affiliations:** Servicio Navarro de Salud-Osasunbidea Hospital Universitario de Navarra Servicio de Cirugía Pediátrica Pamplona Navarra España

## Sra. Editora:

El *pectus excavatum* es la deformidad torácica más frecuente y se presenta con una incidencia de 1 por cada 400-1.000 nacidos vivos^1^. La mayoría de los casos son asintomáticos y no precisan tratamiento alguno. No obstante, la cirugía puede estar indicada cuando existe un *Pectus Severity Index* >3,25, compresión cardiaca o valvulopatía secundaria, pruebas respiratorias con patrón restrictivo, o repercusión psicosocial significativa^2^. La técnica quirúrgica correctiva más extendida es el procedimiento de Nuss o MIRPE (*Minimmally Invasive Repair of Pectus Excavatum*)^1^.

**Figura 1:**
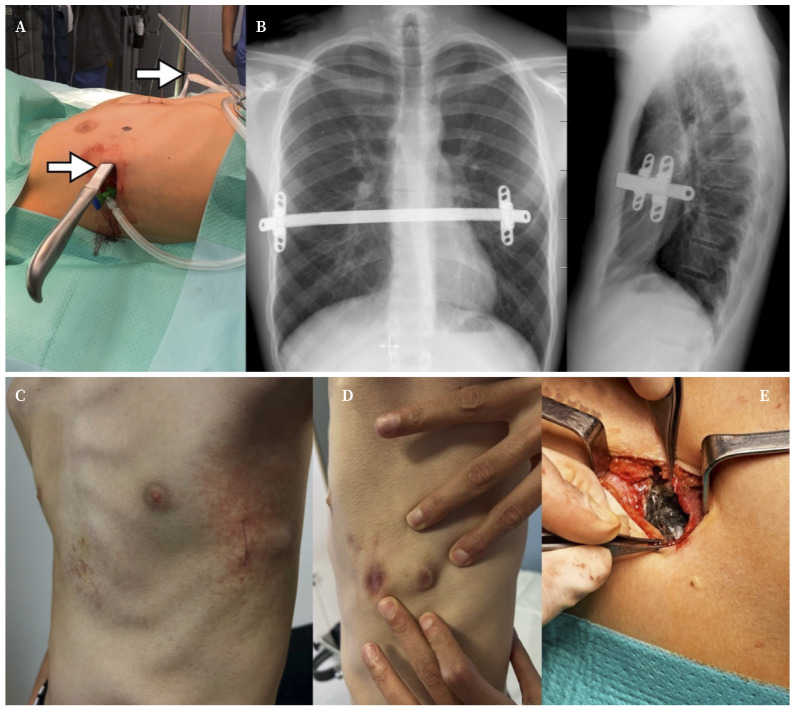
A. Punto de entrada y salida (flechas) del *pectus introducer* o “sable” de disección retroesternal a través del mediastino anterior; en este trayecto se alojará posteriormente la barra metálica. B. Radiografía de tórax frontal y lateral con barra y estabilizadores correctamente posicionados. En el control preoperatorio antes de la retirada del material se aprecian diferentes complicaciones postoperatorias como reacciones cutáneas torácicas (C), reacción a cuerpo extraño al material implantado (D) y metalosis en los tejidos adyacentes al estabilizador de la barra (E).

En este procedimiento se introduce una barra metálica a través del mediastino en situación posterior al esternón, a fin de elevarlo y corregir la depresión de la pared torácica (Fig. 1). La barra colocada en nuestros pacientes es de acero inoxidable (1.4441 ASTM F 138; MedXpert, Alemania). Sus principales componentes, además del hierro, son el cromo (18%) y el níquel (15%). Otros elementos son molibdeno (3%), manganeso (2%) y varios metales y semimetales (silicio, cobalto, cobre y aluminio, cada uno <1%).

El material de osteosíntesis permanece en su lugar durante aproximadamente dos años, periodo tras el cual es retirado.

A lo largo de nuestra experiencia hemos identificado diversas complicaciones postoperatorias tras el implante de la barra metálica, fundamentalmente reacciones cutáneas, serositis y metalosis en el área de implantación, documentada como coloración grisácea de los tejidos debido a la liberación de metales (Fig. 1).

Nuestra hipótesis es que estas complicaciones podrían estar relacionadas con la sensibilización que producen las partículas metálicas liberadas por la barra.

Con esta finalidad, queremos dar a conocer nuestros resultados preliminares tras la instauración en octubre de 2024 de un protocolo de detección de metales en pacientes que se someten a cirugía para retirada de barra de Nuss en el servicio de Cirugía Pediátrica del Hospital Universitario de Navarra (Pamplona, España).

Se realiza un estudio de sensibilización cutánea a metales durante el preoperatorio y, antes de trasladar el paciente al quirófano para la retirada de la barra, se miden los niveles de níquel, vanadio, molibdeno y cromo en orina. Estas pruebas se llevan a cabo el primer día postoperatorio y se repiten a los tres meses de la retirada del material de osteosíntesis.

Durante este periodo de tiempo hemos intervenido cinco pacientes (tres mujeres y dos hombres) con edades entre 14 y 16 años. De estos cinco, una joven presentó una reacción cutánea alrededor de las heridas siete semanas después de implantar la barra metálica, que mejoró con corticoide tópico; desafortunadamente, esta paciente había desestimado la realización de los estudios de sensibilización preoperatoria.

El principal metal alterado fue el níquel; en los pacientes 1, 3 y 5 se observó una elevación de niveles tras retirar la barra, mientras que en las pacientes 2 y 4 los valores disminuyeron progresivamente tras la extracción del material. Los niveles de molibdeno se elevaron en el paciente 1 para posteriormente disminuir, mientras que en los pacientes 3 y 5 los niveles aumentaron a los tres meses de retirada la barra. El cromo solo mostró niveles elevados en el postoperatorio inmediato en las pacientes 4 y 5, pero estos niveles se normalizaron a los tres meses de retirar el implante metálico. Ningún paciente mostró alteraciones en los niveles de vanadio (Tabla 1).

**Tabla 1:** Características demográficas, clínicas y analíticas de la muestra inicial estudiada

	Pacientes				
	1	2	3	4	5
*Sexo*	Masculino	Femenino	Masculino	Femenino	Femenino
*Edad (años)*	16	15	16	16	14
*Tiempo con la barra (días)*	884	912	930	896	1029
*Complicación*	No	Dermatitis periincisional	No	Metalosis tisular	Metalosis tisular
Sensibilización cutánea preoperatoria	Negativa	No realizada	Negativa	Negativa	Negativa
Níquel (µg/L) D0	2,4	9,2	3,6	6,7	5,2
VR: 0-5,0 D1	1,8	7,4	6,6	5,8	22,8
3M	5,9	5,3	3,7	1,7	11,5
Vanadio (µg/g creatinina) D0	0,19	0,19	0,29	0,29	0,39
VR: <1,0 D1	0,29	0,39	0,29	0,59	0,29
3M	0,19	0,19	0,19	0,29	0,19
Molibdeno (µg/L) D0	122	59	81	112	71
VR: 10-124 D1	17	20	11	30	41
3M	95	92	135	0	234
Cromo (µg/L) D0	2	2,9	2,7	9,5	1,1
VR: <7,5 D1	4,2	6,3	5,6	8	8,2
3M	1,7	2,4	2,5	2,4	1,8

VR: valor de referencia; D0: preoperatorio; D1: primer día postoperatorio; 3M: tres meses postoperatorios; los parámetros en negrita hacen referencia a niveles del metal por encima del valor de referencia.

La incidencia de alergia a metales descrito tras la colocación de barras metálicas durante el procedimiento de Nuss oscila entre el 0,5 y el 6,4%, y está causada por los metales que componen el implante^3^; los síntomas más frecuentes incluyen fiebre y lesiones cutáneas similares a las de la dermatitis alérgica. Solo la paciente 2 presentó una dermatitis siete semanas después de la colocación de la barra en el área cutánea que había estado en contacto con el material metálico. Estos hallazgos son congruentes con una posible reacción de hipersensibilidad tipo IV mediada por células Th CD4+, que aparece generalmente en los primeros 40 días después de la cirugía, aunque puede aparecer durante los 140 días postoperatorios^4^; como ha ocurrido en la paciente 2. Es importante tener presente la posibilidad de la reacción de hipersensibilidad y no dar por sentado que los síntomas corresponden a una infección postoperatoria. En esta situación, el tratamiento con histamínicos y corticoides orales suele ser efectivo en la mayoría de los casos, aunque algunos pacientes precisan retirada de la barra y reemplazo con una de titanio, que es más costosa y menos flexible^3^^,^^4^. Esto ratifica la importancia de la valoración alergológica preoperatoria para evitar posibles sensibilizaciones que puedan condicionar el implante de otros dispositivos o prótesis que el paciente pueda requerir posteriormente, lo cual es particularmente importante en la población pediátrica teniendo en cuenta su elevada expectativa de vida. También debe tomarse en consideración que los hombres, las personas con dermatitis atópica y aquellas con determinados polimorfismos genéticos tienen mayor riesgo de hipersensibilidad, y que la alergia puede aparecer en aproximadamente el 1,2% de los pacientes con test preoperatorio negativo^5^.

En nuestra cohorte el principal metal alterado fue el níquel; de hecho, los tres niños presentaban elevación de sus niveles tras retirar la barra, lo cual podría estar relacionado con la movilización del material durante la extracción quirúrgica o con la liberación mantenida desde los tejidos impregnados. No obstante, es difícil establecer un patrón en su comportamiento, ya que en algunas adolescentes (2 y 4) los valores urinarios de níquel disminuyeron progresivamente tras la retirada del material de osteosíntesis. Esto último es compatible con la descripción de la variación de los niveles de níquel urinario descrita por Fortmann y col^6^, donde los niveles preoperatorios (1,8 µg/L) ascendieron al año de colocar la barra (6 µg/L) y disminuyeron progresivamente tras su retirada (1,09 µg/L a los 12 meses y 1,01 µg/L a los 30).

El cromo es otro de los elementos frecuentemente involucrados y que también ha mostrado un aclaramiento progresivo tras retirar la barra. Se ha descrito un aumento significativo de los niveles de cromo en sangre cuando se emplean estabilizadores frente a cuando no se utilizan^7^.

Aunque estos resultados son preliminares, nuestros hallazgos son congruentes con lo descrito en grandes series y deben tenerse en cuenta a la hora de indicar el implante de dispositivos metálicos que potencialmente produzcan sensibilización, como es el caso de la cirugía de Nuss en el *pectus excavatum*. Creemos importante alertar sobre este problema ya que, años después de la cirugía, los pacientes pueden presentar reacciones graves tras implantes médicos críticos (*stents*, válvulas, fracturas, etc.), con repercusiones vitales. En nuestro centro, se han tomado las siguientes medidas: 1) restringir la corrección de las deformidades torácicas solo en jóvenes con repercusión clínica o psicológica demostrable; 2) cribar a jóvenes con sensibilización previa a metales o dermatitis atópica para utilizar con ellos implantes de titanio; y 3) incluir información preoperatoria sobre las eventuales consecuencias de la exposición al metal del material de toracoplastia.
